# Unveiling the acute neurophysiological responses to strength training: An exploratory study on novices performing weightlifting bouts with different motor learning models

**DOI:** 10.5114/biolsport.2024.133481

**Published:** 2023-12-18

**Authors:** Achraf Ammar, Mohamed Ali Boujelbane, Marvin Leonard Simak, Irene Fraile-Fuente, Nikolas Rizzi, Jad Adrian Washif, Piotr Zmijewski, Haitham Jahrami, Wolfgang I. Schöllhorn

**Affiliations:** 1 Department of Training and Movement Science, Institute of Sport Science, Johannes Gutenberg-University Mainz, Mainz, Germany; 2 Interdisciplinary Laboratory in Neurosciences, Physiology and Psychology: Physical Activity, Health and Learning (LINP2), UFR STAPS (Faculty of Sport Sciences), UPL, Paris Nanterre University, Nanterre, France; 3 Research Laboratory, Molecular Bases of Human Pathology, LR19ES13, Faculty of Medicine of Sfax,University of Sfax, Sfax 3029, Tunisia; 4 High Institute of Sport and Physical Education, University of Sfax, Tunisia; 5 Research Unit: “Physical Activity, Sport, and Health”, UR18JS01, National Observatory of Sport, Tunis 1003, Tunisia; 6 Sports Performance Division, National Sports Institute of Malaysia, Kuala Lumpur, Malaysia; 7 Jozef Pilsudski University of Physical Education in Warsaw, Warsaw, Poland; 8 College of Medicine and Medical Sciences, Arabian Gulf University, Manama, Kingdom of Bahrain; 9 Government Hospitals, Manama, Kingdom of Bahrain

**Keywords:** EEG, Brain activity, HRV, Autonomic nervous system, Olympic snatch, Exertion, Mental demand

## Abstract

Currently, there is limited evidence regarding various neurophysiological responses to strength exercise and the influence of the adopted practice schedule. This study aimed to assess the acute systemic effects of snatch training bouts, employing different motor learning models, on skill efficiency, electric brain activity (EEG), heart rate variability (HRV), and perceived exertion as well as mental demand in novices. In a within-subject design, sixteen highly active males (mean age: 23.13 ± 2.09 years) randomly performed snatch learning bouts consisting of 36 trials using repetitive learning (RL), contextual interference (blocked, CIb; and serial, CIs), and differential learning (DL) models. Spontaneous resting EEG and HRV activities were recorded at PRE and POST training bouts while measuring heart rate. Perceived exertion and mental demand were assessed immediately after, and barbell kinematics were recorded during three power snatch trials performed following the POST measurement. The results showed increases in alpha, beta, and gamma frequencies from pre- to post-training bouts in the majority of the tested brain regions (p values ranging from < 0.0001 to 0.02). The CIb model exhibited increased frequencies in more regions. Resting time domain HRV parameters were altered following the snatch bouts, with increased HR (p < 0.001) and decreased RR interval (p < 0.001), SDNN, and RMSSD (p values ranging from < 0.0001 to 0.02). DL showed more pronounced pulse-related changes (p = 0.01). Significant changes in HRV frequency domain parameters were observed, with a significant increase in LFn (p = 0.03) and a decrease in HFn (p = 0.001) registered only in the DL model. Elevated HR zones (> HR zone 3) were more dominant in the DL model during the snatch bouts (effect size = 0.5). Similarly, the DL model tended to exhibit higher perceived physical (effect size = 0.5) and mental exertions (effect size = 0.6). Despite the highest psycho-physiological response, the DL group showed one of the fewest significant EEG changes. There was no significant advantage of one learning model over the other in terms of technical efficiency. These findings offer preliminary support for the acute neurophysiological benefits of coordination-strength-based exercise in novices, particularly when employing a DL model. The advantages of combining EEG and HRV measurements for comprehensive monitoring and understanding of potential adaptations are also highlighted. However, further studies encompassing a broader range of coordination-strength-based exercises are warranted to corroborate these observations.

## INTRODUCTION

A debate on the most effective motor learning theories for optimizing skill acquisition and neurophysiological adaptation is often “heated” and recurring topic among sports scientists, physical education teachers, and coaches. The most discussed motor learning approaches in sports within this context include repetitive learning (RL), variability of practice (VP), contextual interference (CI), and differential learning (DL), although only the RL and DL have been designed for the field of sports [1].

Historically, the RL model, initially popularized by psychologists’ animal experiments, is rooted in the early theories of learning, and often associated with early behaviorist principles of learning. The RL model emphasized the importance of repeating correct trials for optimal skill acquisition. The belief was that through repetition of correct movements, the motor system becomes more efficient, and the learner can perform the specific skills with greater precision and accuracy. However, over time, this theory was challenged. Initially, just following the theory of behaviorism, there was a primary focus on inducing changes in movement behaviors by manipulating external stimuli, including variations in instructions, equipment, environmental factors, etc. [1]. Subsequently, in the second half of the 20^th^ century, the emergence of other models, such as the VP and CI, introduced a shift of variations from the system’s environment toward the system itself, represented by the learner. Both models involve task differentiation, with the VP model varying only the variable parameters, such as absolute timing, absolute forces, etc. [2], while the CI model extends this differentiation to “invariants” (i.e., the generalized motor programs (GMP)) for learning multiple movements in parallel and the schedule (i.e., temporal structuring of the learning process) [3, 4].

More recently, in the late 1990s, the DL model emerged [5, 6], asserting that effective learning requires the more general introduction of differences facilitated by the incorporation of stochastic perturbations or noise [7, 8] that is finely tuned to the learner’s individual and situational characteristics during the learning process [1, 9]. The origin of this model was derived from a variety of other recent research areas. These research areas primarily include (i) the study of individuality in whole-body movements and their non repeatability [10–12]; (ii) principles related to system dynamics, involving the direct or indirect amplification of observed fluctuations to destabilize the system and initiate controlled or guided self-organizing learning processes; (iii) the application of this approach to large degrees of freedom (DGF) movements and the integration of its principles with other models such as CI [1]; and (iv) the utilization of machine learning, specifically artificial neural network (ANN) training methods for pattern recognition, recently used as a valuable tool for quantifying similarities and classifying skills [13–16].

While the RL model aims to map changing behavior/thinking by repetitively imitating a role model, the DL model, in its most extreme interpretation, involves no repetition and no augmented feedback. In between, the CI model originally involves learning a single movement through repetitions that are interleaved with other movements [17–19]. Meanwhile, the CI model is most often applied to the parallel learning of multiple movements (i.e., more than one fine motor skill is practiced in either random or serial order) but considers movement and target errors as destructive to learning progress. In contrast, DL suggests that only differences (facilitated by adding stochastic perturbations during the acquisition process) allow learning to occur and considers errors, which always occur during RL, as essential fluctuations that have a constructive influence on learning through a real self-organizing process where no concrete information about the solution is provided [5, 6, 20, 21].

In studies investigating the CI effect, structured practice setting usually involves two primary forms, namely, blocked and random practices. In blocked practice, learners perform all trials of a task before transitioning to a different task with diverse variable parameters according to the VP model. This approach minimizes the variability of the invariants and allows for repeated task performance [22]. In contrast, random practice involves interleaving tasks with different GMPs in an unpredictable order, with tasks rarely repeated in consecutive trials [22]. The paradoxical two effects of CI assume that random practices hinder performance during the acquisition phase while providing more permanent benefits during the retention or learning phase compared to blocked practice [3, 4].

The advantages of the second effect, random practice in the retention phase, were most often attributed to either increased elaboration, assumed to involve learners in deeper and more elaborate information processing [23], or increased forgetting and reconstruction, which requires extra retrieval practice and reconstruction of tasks’ plans of action [24, 25]. Based on both theoretical positions, during random practice, a more detailed and permanent representation of the task is being memorized, which may be the origin of the enhanced retention performance. Regarding the reduced likely benefits of a random schedule during the acquisition phase, these theoretical positions rely on cognitive load theory [26], suggesting overloading the capacity of working memory during random practice to hinder performance during the acquisition phase, causing the first effect.

Taking into consideration the abovementioned cognitive-psychological explanations regarding the interference phenomenon, further overloaded cognitive abilities are expected following DL compared to CI practice because of higher variety of movements in the DL acquisition sessions. According to the overload of working memory [26], one should anticipate relatively modest improvements in newly acquired skills during the acquisition phase following the DL compared to the CI schedule. Moreover, in the context of DL, the putative correct skill is executed only once, and since the added noise corresponds to erroneous movements, one can anticipate generally poor learning outcomes. Nevertheless, previous studies on DL contradict the cognitive overload theory and non-erroneous movements in the CI model and provide evidence supporting the superiority of stochastic DL training, already during the acquisition phase, over repetition-based training in the context of learning a single movement [13, 27–32], as well as over CI learning in the context of learning multiple skills simultaneously in football [32] and volleyball [27, 28, 33].

The failure of the CI-related cognitive load theory to corroborate the superiority of DL over CI acquisition practice can be attributed to the fact that the working memory model relied upon by CI was originally developed only for sequential, visual-spatial content [34], a domain where the model still demonstrates its highest reliability. Therefore, a comprehensive explanation based on reliable neurophysiological measurements is still needed to elucidate how DL practice exhibited the largest short-term benefits while being assumed to further overload the learner’s cognitive abilities compared to CI and RL.

Neurophysiological evidence from MRI and EEG research supports the theoretical constructs of the different motor learning models. Findings from MRI studies provided evidence for corroborating different brain activations during blocked vs. randomized practice [35, 36]. However, this has only been observed in movements with few DGFs due to restrictions associated with the available measurement devices. Additionally, these studies only compared different levels of CI practice, with no emphasis on comparative measurements between CI and DL. Comparing brain activity immediately after RL, CI, and various forms of DL practice in badminton, previous EEG-based studies have shown distinct patterns [37, 38]. After CI practice, an increase in gamma and beta frequencies has been recorded in the frontal lobe, indicating the engagement of executively controlled cognitive processes [38]. In contrast, following all forms of DL practice, increases in theta and alpha activities were obtained in somatosensory and motor areas, while activation in the frontal cortex was downregulated toward increased theta and decreased beta and gamma frequencies [38]. This shift in the anterior brain areas suggests that the CI model may induce increased cognitive stress in badminton learners when compared to DL [37, 38]. In this context, recent reports have suggested that the wide range of skill variations in DL acts as a mitigating factor, reducing the risk of medium- and long-term frontal lobe overload, which in turn aids in the motor learning of movements with a high DGF [1, 38, 39]. However, additional neurophysiological comparative studies are necessary to validate this explanation across a broader spectrum of sports skills. On the other hand, combining high CI practice (random) with complex and highly demanding motor tasks could exacerbate cognitive load, thereby reducing the likely benefits of random practice. Again, such assumptions need to be proven using reliable neurophysiological measurements during the acquisition of highly demanding sport skills with high DGF.

According to Cioroslan [40], Olympic weightlifting is a sport of outstanding neuromuscular coordination, fine kinesthetic perception, agility, and the ability to perform explosive movements in a specific line of technique with maximum accuracy. Additionally, Olympic weightlifting exercises, acknowledged as the most intense and demanding strength exercises, are among the few exercises with high DGF that involve the entire body musculature by activating complete muscle chains [41]. It is worth noting that the majority of neurophysiological studies have concentrated on endurance-based sports, leaving a notable gap in our understanding regarding brain activity responses to strength-based exercises [42], such as weightlifting (e.g., snatch, clean and jerk). These exercises present an excellent opportunity to empirically investigate motor learning related assumptions discussed above, using reliable neurophysiological measurements. In this context, electroencephalogram (EEG) is a commonly used tool in research to monitor the brain’s electrical activity. It effectively provides insights regarding brain waves in different frequency bands, including the theta (4–7.5 Hz), alpha (8–13 Hz), beta (13–30 Hz), and gamma (30–40 Hz) signals, which are the crucial indicators for understanding neurophysiological states or responses to the different motor learning tasks. Furthermore, recent studies have emphasized the role of the autonomic nervous system in regulation of mental stress level, cognitive functioning, and motor performance [43–45]. Indeed, a greater heart rate variability (HRV) has been associated with reduced stress level and enhanced cognitive and motor functioning [43, 45]. Therefore, it is pertinent to incorporate HRV parameters as part of neurophysiological measurements within the domain of motor control.

Nonetheless, only a few studies have concurrently assessed both EEG and HRV while also controlling the rates of perceived exertion (RPE) during exercises with high DGF performed following different motor learning approaches. For instance, John and Schöllhorn [46, 47] compared the effects of RL and DL approaches, including instructed and self-created variable learning, for learning rope skipping movements on multiple neurophysiological responses. The authors suggested that combining rope skipping with additional cognitively demanding tasks in the form of the DL approach might lead to short-term cognitive overload. They also emphasized the promising, yet inconclusive advantages of DL over RL and highlighted the need for further research, including the CI approach, to comprehensively explore the reciprocal influence of cardiac and central nerve strains in greater detail.

In view of the limited evidence regarding the impact of different motor learning approaches on technical efficiency and the accompanying neurophysiological aspects, specifically in response to strength-based exercise, the present study aimed to investigate the acute systemic effects of three different motor learning models, namely, the RL, CI, and DL, on the technical efficiency the Olympic snatch, along with EEG brain activity and HRV responses in novice learners. This research seeks to provide a more comprehensive understanding of the complex interplay between cognitive load, practice variations, and brain and autonomic nervous system activation patterns. Such insights are crucial for customizing training interventions and optimizing skill acquisition and performance in diverse motor learning contexts.

We hypothesize that, due to the increased mental effort involved in the execution of various coordination tasks, the DL approach would engage more regions of the cortex than RL and CI, particularly at the somatosensory and motor areas. In contrast, CI would specifically involve executive processing in the frontal area. Additionally, we hypothesize that DL would lead to a lower activation of the sympathetic system through a decrease in the high-frequency band and an increase in the low-frequency band, as well as a greater perceived exertion and mental demand.

## MATERIALS AND METHODS

### Participants

The sample size was calculated *a priori* based on procedures suggested by Beck [48] and using the software G∗power [49]. Values were set at 0.05 for α and 0.95 for power. Based on the studies of John & Schöllhorn [46] and discussions between the authors, the effect size was set to be 0.5 (medium effect). The minimum needed sample size for this study was 10. Sixteen highly active males were recruited to voluntarily participate in this study. After receiving a description of the protocol, potential risks, and benefits of the study, participants gave their written consent to participate in this investigation. The criteria for participant inclusion in the present study are as follows: all participants should be aged between 18 and 29 years old, male, right-handed as assessed by the Edinburgh Handedness Inventory, and should have at least 2 years of experience in fitness and/or CrossFit club (i.e., including at least 6 months of performing barbellbased exercises). Exclusion criteria included previous experience in Olympic weightlifting, current or a history of neurological and/or cardiovascular impairment, eye disorders, psychiatric illnesses, orthopedic diseases, and/or muscular disorders, and the intake of medication that may have influenced EEG brain activity and/or HRV.

The study was conducted according to the Declaration of Helsinki; all subjects were naive to the purpose of the study and were coded with numbers for the anonymity of personal data. The study was approved by the local ethics committee of Faculty 02: Social Sciences, Media, and Sport at Johannes Gutenberg University of Mainz.

### Experimental design

A randomized within-subject design was employed to assess the short-term effects of four different motor learning approaches. After a familiarization session, participants reported to the laboratory on four separate occasions, with at least a one-week washout period in between. In a randomized order, during each test session, one single motor learning approach was conducted in a single training bout. This training bout consisted of 36 trials of power-snatch derivates according to one of the four tested motor learning approaches: RL, CI blocked (CIb) or CI serial (CIs), and DL, with a standardized duration of ≈ 3 minutes. The sequence of motor learning approaches was randomized, and all test sessions were performed at the same time of day (in the afternoon, as previously suggested by Ammar et al., [50, 51]) to minimize the effect of diurnal biological variations [52]. The measurements were carried out under laboratory conditions. Changes in brightness, volume, and temperature were standardized and kept to a minimum.

**FIG. 1:**
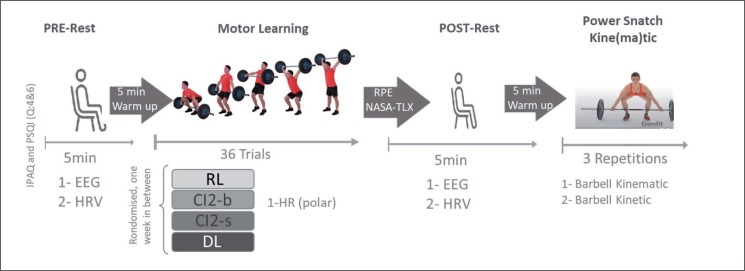
Study Design.

The procedure for each testing session is detailed in Figure 1. Upon arrival at the laboratory, participants completed the short form of the International Physical Activity Questionnaire (IPAQ-SF) and questions 4 and 6 of the Pittsburgh Sleep Quality Index (PSQI). These questionnaires were used to assess their physical activity behaviors and sleep duration and quality over the previous 7 days prior to testing. Spontaneous EEG activity and electrocardiography (ECG) HRV were recorded in a sitting position with eyes open before (PRE-rest, duration: 5 min) and after (POST-rest, duration: 5 min) each training session. Ratings of perceived exertion (RPE) and perceived physical and mental demand (NASA Task Load Index_NASA-TLX) were assessed immediately following each training bout. Additional HR was recorded using the Polar H10 HR monitor during each training bout. To determine the effects of the different learning approaches on technical efficiency, barbell kinematics were recorded during three-power snatch trials performed following the POST-rest measurement (Figure 1).

### Motor Learning Approaches

The training bouts for the RL approach consisted of 36 repetitions of power snatch. The training bouts of both CI approaches (CIb and CIs) included the power snatch and two power snatch variations: the snatch power jerk (known to improve overhead strength, stability, balance, and barbell control in the catch phase; Soriano et al. [53]) and the high pull snatch (known to improve strength, speed, power, posture, and balance in the extension of the snatch; [54]). Specifically, the training bout of the CIb approach consisted of practising high pull snatch (A), snatch power jerk (B), and power snatch (C) in blocked order: 12 reps (A), 12 reps (B), and 12 reps (C) for a total of 36 trials. The training bout of the CIb approach consisted of practising high pull Snatch (A), snatch power jerk (B), and power snatch (C) in serial order: ABC × 12, with a total of 36 trials.

These three movements were also practiced in serial order (ABC × 12 with a total of 36 trials) during the training bout of the DL approach. However, variations in different movement/environmental parameters, such as foot starting position (for A, B, and C), barbell starting position (for A and C), final positions (for B), eyes closed (for A, B, and C), and/or unstable surface (using the aeris® muvmat [55] for A, B, and C), have been adopted to generate additional movement variability and increase fluctuations. In general, DL training applies movement variations and increased fluctuations to make the system unstable and thereby foster a self-organized learning process by achieving greater effects on brain activity [56]. In addition to the referential condition (no additional movement variability), three levels of movement variability are identified in the present power snatch DL approach: level 1: variation in only one movement/surrounding parameter; level 2: variation in 2 movement/surrounding parameters; and level 3: variation in 3 movement/surrounding parameters.

To homogenize physical (cardiac) exertion in all motor learning approaches according to exhaustion influence on brain activity [57, 58] as well as on the cardiovascular system [59, 60], a ninesecond interblock (12 repetitions) rest period was adopted in the RL and both CI conditions (total rest period of 18 seconds during the 36 trials), and a six-second inter-set rest period was adopted in the DL condition (total rest period of 18 seconds during 36 trials). This procedure allowed us to adopt a standardized training bout duration of ≈ 3 minutes. Furthermore, the training bouts for all approaches were performed using the same empty barbell (10 kg).

### Measurements

#### Physical activity and sleep pattern

The IPAQ-SF has been extensively validated in different cultures and populations [61]. The total weekly PA (MET-min · week-1) was estimated by multiplying the reported weekly time for each IPAQ-SF item (vigorous intensity, moderate intensity, and walking) by their respective metabolic equivalent of task (MET) values [62]. We utilized the original MET values recommended by the official IPAQ guidelines for young and middle-aged adults (18–65 years old): vigorous PA = 8.0 METs, moderate PA = 4.0 METs, and walking = 3.3 METs. Following the IPAQ scoring protocol, participants in the study were categorized into three groups based on their MET–min/wk, which represents the cumulative sum of walking, moderate-intensity physical activities, and vigorous-intensity physical activities: lowly active (< 600 MET–min/wk), moderately active (600 MET–min/ wk ≤ PA < 3000 MET–min/wk), and highly active (≥ 3000 MET–min/wk) (http://www.ipaq.ki.se).

### Electroencephalogram (EEG) measurement

#### EEG Data Acquisition

**FIG. 2:**
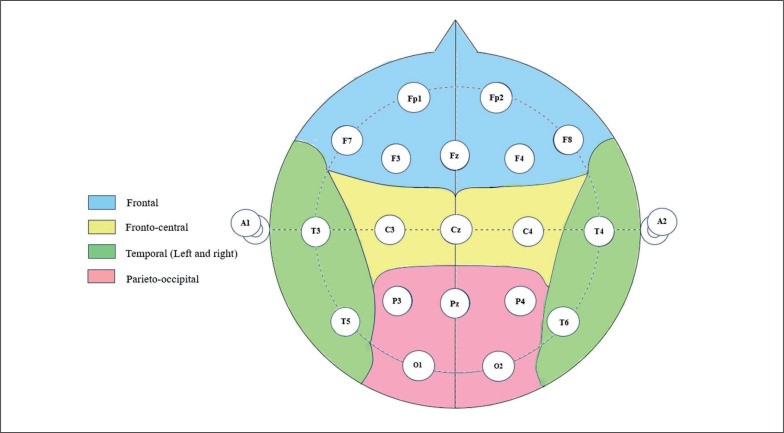
Scalp location.

Spontaneous resting EEG was assessed by means of the Micromed SD LTM 32 BS EEG system (Venice, Italy) with a sampling rate of 1024 Hz. This EEG system is composed of 19 electrodes, including Fp1, Fp2, F7, F3, Fz, F4, F8, T3, C3, Cz, C4, T4, T5, P3, Pz, P4, T6, O1, and O2, organized according to the international 10–20 system. The 10–20 system is based on the relationship between the electrode location and the subregions of the cerebral cortex. Each point on the system indicates a possible position for the electrode, with a letter to identify the lobes and a number to identify the hemisphere. The 10–20 system ensures accurate electrode placement, enabling researchers to obtain reliable EEG signals. Resting EEG signals were continuously registered before (5 min) and after (5 min) the different training bouts in a sitting position. For all EEG measurements, a homogeneous and low impedance (< 10 kΩ) of the electrodes at all points was sought. Five scalp locations (Figure 2), frontal “F” (Fp1, Fp2, F7, F3, Fz, F4, F8), central “C” (C3, Cz, C4), temporal (left (T-L): T3, T5; right (T-R): T4, T6), and parietal-occipital “P/O” (P3, Pz, P4, O1, and O2), were chosen to gather the signals from the 19 electrodes. The conduction of brain activity was unipolar, with grounding on the nose. Furthermore, a two-channel electrooculogram with electrodes at the medial upper and lateral orbital rims of the right eye was applied. Data were recorded by means of commercially available software (SystemPlus Evolution—Micromed, Venice, Italy).

### EEG data preprocessing and analysis

Once the raw signals were recorded, we exported them to MATLAB (EEGLAB-Toolbox 2019 from the University of California, San Diego) for processing.

The data were first visually inspected to remove noisy segments related to electrode placement or technical disturbances. A bandpass filter was set from 2–40 Hz. Independent component analysis (ICA) was performed to deduce recurrent movement from the signal, such as eye blinks, muscle activity, or channel noise. Impaired electrodes were interpolated using a spherical spline interpolation method [63]. A final visual inspection of the time series and the power spectrum was conducted to remove any remaining artifacts.

Spectral power was calculated for each testing session using fast Fourier transformation with a Hanning window, a window size of 4,096 samples (4 s), and a window overlap of 50% for theta [3.5–7.5 Hz], alpha [7.5–12.5 Hz], beta [12.5–30 Hz], and gamma [30–40 Hz]. Band ranges were chosen according to previous studies that involved movement and EEG [38, 47, 55, 64]. The power spectrum was computed for each test session.

### Heart rate and heart rate variability (HRV) measurements

Before and immediately after the different training bouts, HRV parameters were measured using a Polar H10 HR monitor with a Pro Strap (Polar Electro Oy, Kempele, Finland). The Polar H10 system measures the electrical signal of the heart in the form of a 1-channel electrocardiogram (ECG) signal using two dry electrodes. From the ECG signal, the R-peaks are detected to derive the RR interval time series. Both the ECG quality [65] and the quality of the derived RR intervals [66, 67] using Polar H10 were previously compared to the gold standard ECG measurement using a 12-channel medical-grade ECG device and found to be excellent.

### HRV measurement and analysis

Prior to the test sessions, the Polar H10 electrodes were moistened with room-temperature water prior to being placed on the xiphoid process of the sternum with the chest strap fitted around the participant’s chest (just below the chest muscles) [55, 68]. The Android app “Polar Sensor Logger” [69] was used to record the RR intervals. Five minutes of resting in an upright sitting position was recorded. The recorded data were later imported and analyzed in Kubios HRV Standard 3.5.0 analysis software [70]. Using Kubios, artifact removal and detrending were performed as necessary preprocessing steps. Therefore, RR intervals that were larger or smaller than a set threshold compared to the local average were corrected by replacing the identified artifacts with interpolated values using cubic spline interpolation. The threshold was adjusted individually but was generally in the range of 0.25–0.35 seconds (low or medium threshold). Afterwards, the smoothness priors detrending method [71] was used to avoid the effect of slow nonstationary trends in the analysis.

Using the preprocessed RR intervals, several time- and frequency-domain parameters were calculated. From the time domain, we chose the most often used parameters for short-term analysis, which are the mean (MeanRR), the standard deviation (SDNN) and the root mean square of successive differences (RMSSD) of the RR intervals. We also analyzed the lowest (HR-min) and highest (HR-max) values, as well as the average of HR values (HR-mean). To calculate the frequency parameters, the time series was resampled to 4 Hz, and the FFT spectrum was derived using Welch’s periodogram. The low frequency (LF, 0.04–0.15 Hz) and high frequency (HF, 0.15–0.4 Hz) power (n.u.) and the LF/HF ratio were computed from the FFT spectrum. Furthermore, we calculated the coefficient of variation (CVNN) by the formula CVNN = 100 * SDNN/MeanRR. The CVNN tries to minimize the mathematical dependence of the standard deviation from the mean through normalization.

### HR zones

HR zones are a useful method for monitoring training intensity [68]. These zones categorize training intensity based on a percentage of the maximum heart rate and are commonly divided into five ranges. For our study, we adhered to the Polar recommendations for each HR zone (https://www.polar.com/blog/running-heart-rate-zones-basics/): Zone 1 (50–60%), Zone 2 (60–70%), Zone 3 (70–80%), Zone 4 (80–90%), and Zone 5 (90–100%). The time spent in each HR zone was calculated as a percentage of the whole training bout time (e.g., spending 1 minute in zone 3 during the three-minute training bouts corresponds to 33.3%). Similar to the HRV, HR zones were controlled using the Polar H10 HR monitor with a Pro Strap (Polar Electro Oy, Kempele, Finland).

### Rating of perceived exertion (RPE)

The RPE was obtained immediately following each training bout using an 11-point scale, with scores ranging from 0 (very, very light) to 10 (very, very hard). An intraclass correlation coefficient (ICC) of 0.83 showed that RPE was a good measure of physical effort. It also had good psychometric properties and a strong correlation with a number of other physiological measures of effort [72].

### Subjective mental workload

The subjective rating of mental workload was assessed using the NASA-Task Load Index scale (NASA-TLX). The NASA-TLX uses six subscales to assess mental workload, including mental demand, physical demand, temporal demand, performance, effort, and frustration [73]. In the present project, we focused on the analysis of mental demand.

### Kinetic and kinematic measures

The three snatch trials performed on a 2.4 × 0.9 m weightlifting platform were recorded using nine synchronized, commercially available infrared cameras (Oqus 300/310+, Qualisys AB, Sweden) positioned around the platform at a distance of ~6 m from the lifting area, and kinematic data were collected at 250 Hz using Qualisys Track Manager 2.7 (Qualisys AB, Sweden). Two reflective markers were attached to the right and left ends of the barbell, and the calibration was executed beforehand using a calibration wand according to the Qualisys manual.

As suggested by Kipp, Harris, and Sabick [74], during postprocessing, the barbell data were filtered by means of a 2^nd^-order Butterworth filter with a cutoff frequency of 20 Hz. Computations of vertical and horizontal barbell displacements were conducted using the average location of the right and left markers on the barbell. The average of both markers provides movement of the bar center and avoids the induction of artifacts associated with asymmetrical movement [75, 76]. In the present study, we focused on the peak vertical displacement of the barbell registered during the turnover phase and on three key horizontal displacement values, which include the maximal horizontal displacement during the first pull (mh1) and the second pull (mh2) and the horizontal displacement from the most forward position to the catch position (DXL or loop) [41, 75–77].

### Statistical analyses

Mean and standard deviation (SD) values were calculated for each variable. The Shapiro-Wilk W-test was used to verify normal distribution of data. A two-way repeated measures analysis of variance (RMANOVA) (i.e., 4 levels [motor learning approaches] × 2 levels [PRE/POST-training]) was used to analyze the EEG and HRV data, and a one-way analysis of variance (RMANOVA) (4 levels [motor learning approaches: RL, CIb, CIs, and DL]) was used to analyze differences between training practices in terms of HR zones during the training bouts, perceived exertion and perceived mental workload, and barbell displacements. When the main effects were found to be significant, post hoc t tests with Bonferroni correction or Wilcoxon tests were used. The equality of variances (homogeneity) was verified using Bartlett’s test. To estimate the meaningfulness of significant differences, effect sizes were calculated as partial eta-squared (ηp2) for the main effects and the interaction between them and as Cohen’s d (d) for the paired comparison. Values of 0.01, 0.06, and 0.13 for partial eta-squared and 0.2, 0.5, and > 0.8 for Cohen’s d represent small, moderate, and large effect sizes, respectively. To estimate the magnitude of significant differences, difference (Δ) or percent difference (Δ (%)) scores were calculated as follows: Δ =postbouts value – prebouts value; Δ (%)=[((post value – pre value))/(pre value)] × 100. The differences between training models in terms of Δ, particularly for EEG and HRV parameters, were analyzed using a one-way RMANOVA (4 levels [motor learning approaches: RL, CIb, CIs, and DL]). Additionally, relationships between EEG and HRV-related parameters at post-training bouts were analyzed using the Pearson correlation coefficient (r). Significance was accepted for all analyses at the level of p < 0.05. Exact p values have been given; results given as ‘‘0.000’’ in the statistics output have been reported as ‘‘ < 0.0005’’.

## RESULTS

### Participants’ characteristics

Sixteen highly active athletes (mean age: 23.13 ± 2.09 years) were recruited to participate in this study. Their average body mass index (BMI) was 24.14 ± 2.17, indicating a normal weight range. All participants reported no chronic diseases or sleep disturbances. They reported a fairly good to very good level of sleep quality. Regarding lifestyle habits, 87.5% were nonsmokers, and 25% abstained from alcohol consumption. Concerning the time spent on all types of sports activities, participants reported weekly averages of 225.9 ± 137.1 minutes in vigorous activity, 108.4 ± 77.9 minutes in moderate activity, and 631.7 ± 629.5 minutes in walking. Overall, they engaged in a weekly average of 966.1 ± 274.5 minutes across all sports activities.

According to the IPAQ-SF, the participants were highly active, with an average of 4325.82 ± 2666.27 MET.

### Resting EEG

The acute effects of the weightlifting training bouts on resting brain activity are presented in Table 1 for theta, alpha, beta, and gamma frequencies.

**TABLE 1:** The acute effect of the weightlifting training bouts on resting brain activity for theta, alpha, beta, and gamma frequencies.

	Pre-Training bouts				Post-training bouts				ANOVA 2 ways		
	CIb	CIs	DL	R	CIb	CIs	DL	R	Interaction Group × Training (F, p value, ηp²)	Group effect (F, p value, ηp²)	Training effect (F, p value, ηp²)
**Theta frequency (N ± SD)**											
F	0.85 ± 2.09	0.87 ± 2.45	1.81 ± 2.14	1.43 ± 2.3	1.22 ± 2.36	1.34 ± 2.35	1.46 ± 2.38	1.28 ± 2.93	(F=1.26, p=0.30, ηp²= 0.06)	(F=0.24, p=0.87, ηp²= 0.01)	(F=0.17, p=0.68, ηp²= 0.003)
C	4.66 ± 2	4.77 ± 2.28	5.27 ± 2.16	4.92 ± 2.39	5.08 ± 2.52	5.04 ± 2.69	5.25 ± 2.72	5.15 ± 2.68	(F=0.32, p=0.82, ηp²= 0.02)	(F=0.10, p=0.96, ηp²= 0.01)	(F=1.09, p=0.31, ηp²= 0.02)
T-L	2.87 ± 2.36	2.93 ± 2.21	3.74 ± 2.59	3.57 ± 2.66	3.22 ± 3.34	3.22 ± 3.01	3.47 ± 3.84	3.72 ± 2.96	(F=0.68, p=0.57, ηp²= 0.03)	(F=0.25, p=0.86, ηp²= 0.01)	(F=0.20, p=0.66, ηp²= 0.003)
T-R	3.08 ± 2.79	3.25 ± 2.63	3.59 ± 2.62	3.36 ± 2.81	3.41 ± 3.03	3.17 ± 3.22	3.78 ± 2.98	3.8 ± 2.95	(F=0.46, p=0.71, ηp²= 0.02)	(F=0.13, p=0.94, ηp²= 0.01)	(F=1.86, p=0.18, ηp²= 0.03)
P/O	5.02 ± 2.37	5.25 ± 2.5	5.55 ± 2.56	5.36 ± 2.62	5.38 ± 2.96	5.59 ± 2.98	5.73 ± 3.09	5.83 ± 2.74	(F=0.11, p=0.96, ηp²= 0.01)	(F=0.10, p=0.96, ηp²= 0.01)	(F=4.54, p=0.04, ηp²= 0.08)
**Alpha frequency (N ± SD)**											
F	-0.16 ± 3.08	-0.45 ± 3.03	0.26 ± 2.92	0.33 ± 3.22	0.58 ± 3.04	0.43 ± 2.74^a^	0.55 ± 2.94	0.67 ± 2.99	(F=1.18, p=0.33 ηp²= 0.06)	(F=0.10, p=0.96, ηp²= 0.01)	(F=17.05, p=0.0001, ηp²= 0.22)
C	4.79 ± 3.35	4.55 ± 3.27	4.97 ± 3.3	4.91 ± 3.48	5.75 ± 3.46^a^	5.4 ± 3.38	5.75 ± 3.27	5.93 ± 3.23^a^	(F=0.17, p=0.91 ηp²= 0.01)	(F=0.06, p=0.98, ηp²= 0.003)	(F=45.89, p < 0.0001, ηp²= 0.43)
T-L	4.24 ± 3.86	4 ± 3.46	4.75 ± 3.54	4.91 ± 3.8	5.05 ± 4.01	4.93 ± 3.6	5.29 ± 4.12	5.54 ± 3.16	(F=0.37, p=0.77, ηp²= 0.02)	(F=0.14, p=0.94, ηp²= 0.01)	(F=24.98, p < 0.0001, ηp²= 0.30)
T-R	4.87 ± 4.37	4.97 ± 3.78	5.11 ± 3.71	5.13 ± 4.11	5.68 ± 4.23	5.24 ± 4.28	5.94 ± 3.38^a^	6.27 ± 3.63^a^	(F=1.05, p=1.95, ηp²= 0.13)	(F=0.07, p=0.98, ηp²= 0.004)	(F=18.49, p < 0.0001, ηp²= 0.36)
P/O	7.55 ± 3.92	7.73 ± 3.86	7.88 ± 3.74	8.05 ± 3.89	8.55 ± 4.04^a^	8.73 ± 3.93^a^	8.93 ± 3.57^a^	9.07 ± 3.42^a^	(F=0.01, p=0.99, ηp²= 0.0004)	(F=0.06, p=0.98, ηp²= 0.003)	(F=61.35, p < 0.0001, ηp²= 0.51)
**Beta frequency (N ± SD)**											
F	-6.28 ± 2.72	-6.1 ± 2.41	-5.46 ± 2.01	-5.49 ± 2.51	-4.95 ± 2.55^a^	-5.13 ± 1.96^a^	-4.69 ± 2.08	-4.79 ± 2.14	(F=1.19, p=0.32, ηp²= 0.06)	(F=0.27, p=0.85, ηp²= 0.01)	(F=53.10, p < 0.0001, ηp²= 0.47)
C	-2.95 ± 2.27	-2.97 ± 2.06	-2.59 ± 1.92	-2.54 ± 2.42	-1.89 ± 2.2^a^	-2.17 ± 2.08^a^	-1.74 ± 2.06^a^	-1.7 ± 2.13^a^	(F=0.28, p=0.83, ηp²= 0.01)	(F=0.16, p=0.92, ηp²= 0.01)	(F=65.27, p < 0.0001, ηp²= 0.52)
T-L	-4.07 ± 2.53	-3.61 ± 2.16	-2.98 ± 1.66	-2.96 ± 2.23	-2.53 ± 2.63^a^	-2.59 ± 2.17	-2.43 ± 2.48	-1.96 ± 2.14	(F=1.30, p=0.28, ηp²= 0.06)	(F=0.49, p=0.70, ηp²= 0.02)	(F=33.15, p < 0.0001, ηp²= 0.36)
T-R	-3.85 ± 2.7	-3.32 ± 2.41	-3.17 ± 1.78	-3.36 ± 2.57	-2.48 ± 2.33^a^	-2.87 ± 2.66	-2.04 ± 1.79^a^	-2.19 ± 2.16^a^	(F=2.37, p=0.08, ηp²= 0.11)	(F=0.22, p=0.88, ηp²= 0.01)	(F=62.25, p < 0.0001, ηp²= 0.51)
P/O	-2.25 ± 2.36	-2 ± 1.96	-2 ± 1.77	-1.82 ± 2.3	-1.15 ± 2.48^a^	-1.27 ± 2.2^a^	-1 ± 2.07^a^	-0.87 ± 1.87^a^	(F=0.80, p=0.50, ηp²= 0.04)	(F=0.08, p=0.97, ηp²= 0.004)	(F=116.54, p < 0.0001, ηp²= 0.66)
**Gamma frequency (N ± SD)**											
F	-9.82 ± 2.72	-9.47 ± 2.24	-8.82 ± 1.85	-8.9 ± 2.38	-7.87 ± 2.56^a^	-7.92 ± 1.88^a^	-7.71 ± 2.35	-7.5 ± 1.88^a^	(F=0.70, p=0.56, ηp²= 0.04)	(F=0.37, p=0.78, ηp²= 0.02)	(F=51.88, p < 0.0001, ηp²= 0.46)
C	-7.59 ± 2	-7.34 ± 1.9	-7.15 ± 1.5	-7.02 ± 2.23	-6.07 ± 2.42^a^	-6.2 ± 1.73^a^	-5.85 ± 2.17^a^	-5.74 ± 1.64^a^	(F=0.23, p=0.87, ηp²= 0.01)	(F=0.22, p=0.89, ηp²= 0.01)	(F=63.51, p < 0.0001, ηp²= 0.51)
T-L	-8.06 ± 2.55	-7.3 ± 2.21	-6.45 ± 1.84	-6.49 ± 2.37	-5.81 ± 3.06^a^	-5.75 ± 2.1	-5.88 ± 3.01	-5.05 ± 2.33	(F=1.61, p=0.20, ηp²= 0.08)	(F=0.80, p=0.5, ηp²= 0.04)	(F=28.39, p < 0.0001, ηp²= 0.32)
T-R	-8.1 ± 2.68	-7.37 ± 2.16	-7.04 ± 1.62	-7.5 ± 2.7	-6.13 ± 2.69^a^	-6.39 ± 2.38	-5.46 ± 2.01^a^	-5.69 ± 1.95^a^	(F=1.11, p=0.35, ηp²= 0.05)	(F=0.48, p=0.70, ηp²= 0.02)	(F=60.19, p < 0.0001, ηp²= 0.50)
P/O	-7.39 ± 2.61	-7.08 ± 1.61	-7.02 ± 1.62	-6.88 ± 2.37	-5.87 ± 2.88^a^	-6.05 ± 2.03^a^	-5.75 ± 2.33^a^	-5.52 ± 1.69^a^	(F=0.41, p=0.75, ηp²= 0.02)	(F=0.14, p=0.94, ηp²= 0.01)	(F=67.26, p < 0.0001, ηp²= 0.53)

a : significant difference from pre to post; Frontal (F), Central (C), temporal left (T_Left), temporal right (T_right) and parieto-occipital (P/O) regions; Contextual interference blocked CIb, Contextual interference serial (CIs), differential learning (DL), Repetitive (R) motor learning models.

### Theta

For the theta frequency range, the two-way ANOVA revealed no statistically significant effects for training, group, or their interaction in all studied regions (F, C, T-L, T-R, and P/O), except for a significant training effect found in the P/O region (p = 0.04). All pairwise comparisons in the post hoc analyses were not significant (Table 1). Similarly, no significant differences among the groups were identified in the Δ pre-post training bouts.

### Alpha

**FIG. 3:**
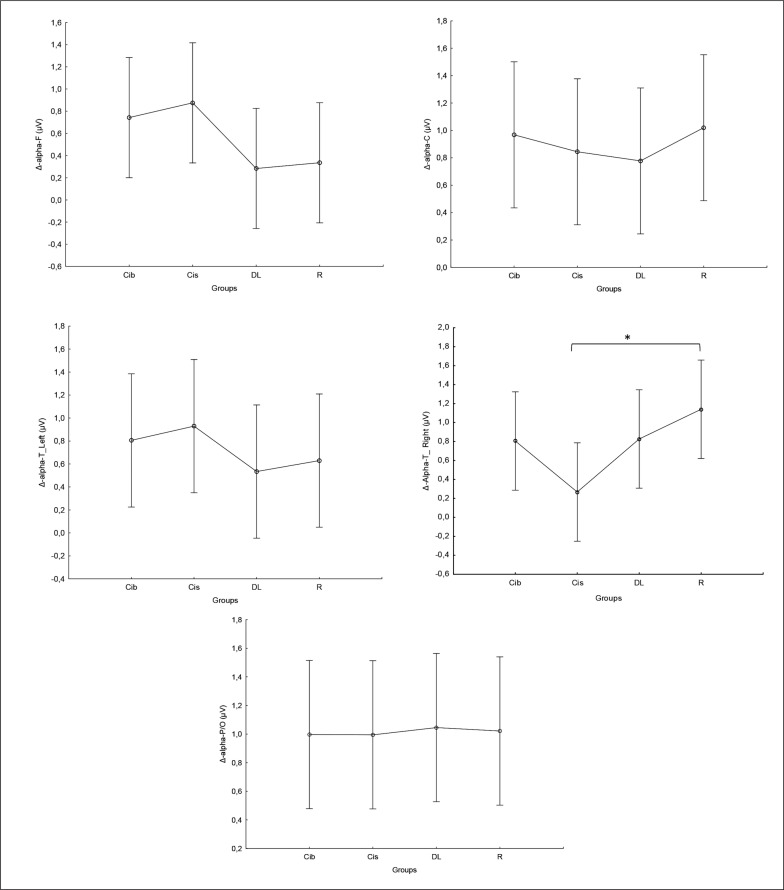
The acute effect of weightlifting training bouts on Δ (pre-post bouts) alpha frequency recorded at different brain regions. a: significant difference between groups at p < 0.05; Frontal (F), Central (C), temporal left (T_Left), temporal right (T_right) and parieto-occipital (P/O) regions; Contextual interference blocked (CIb), Contextual interference serial (CIs), Differential Learning (DL), Repetitive (R) motor learning models.

Regarding the alpha frequency, there was no significant interaction (training × group) and no significant main effect of the group across all parameters. However, statistically significant main effects of training were found in all examined regions (F, C, T-L, T-R, and P/O). Particularly, significantly higher values during the post-training bouts compared to the pre-training bouts were found in CIb in the C (p = 0.02) and P/O (p = 0.01) regions, in CIs in the F (p = 0.05) and P/O (p = 0.02) regions, in DL in the T-R (p = 0.05) and P/O (p = 0.004) regions, and in the R group in the C (p = 0.01), T-R (p = 0.001) and P/O (p = 0.01) regions (Table 1). Regarding the Δ pre-post training bouts, a significantly lower increase was observed in CIs compared to the RL group in the T-R region (p = 0.02). In contrast, in the frontal region, the CI group showed a trend toward nonsignificant increases compared to DL and RL (ES = 0.5, Figure 3).

### Beta

**FIG. 4:**
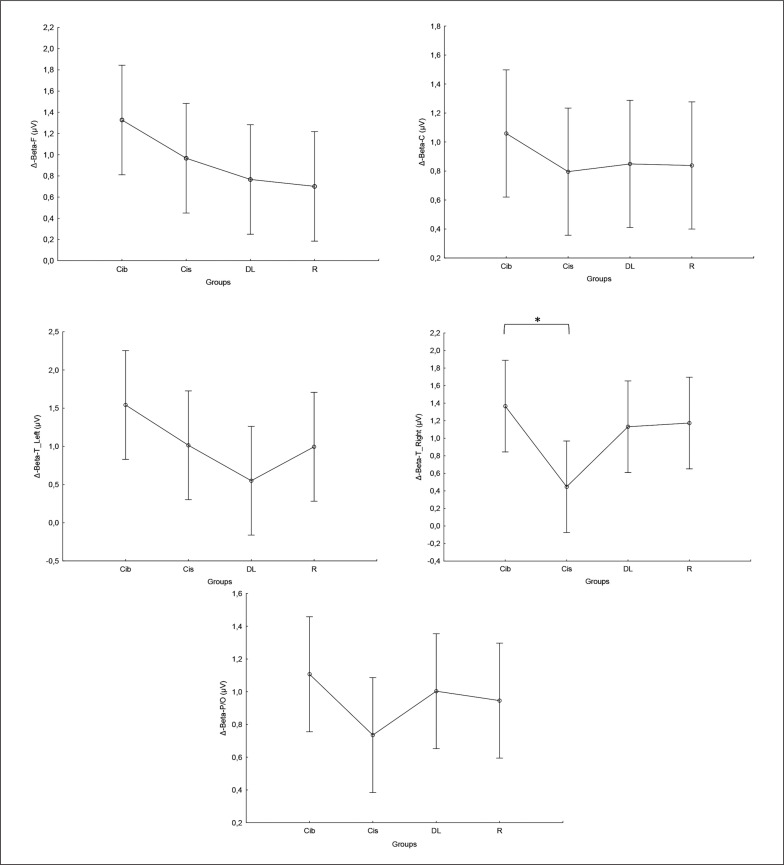
The acute effect of weightlifting training bouts on Δ (pre-post bouts) Beta frequency recorded at the frontal, fronto-central, temporal (Left and right) and parieto-occipital regions. a: significant difference between groups at p < 0.05; Frontal (F), Central (C), temporal left (T_Left), temporal right (T_right) and parieto-occipital (P/O) regions; Contextual interference blocked CIb, Contextual interference serial (CIs), Differential Learning (DL), Repetitive (R) motor learning models.

Similarly, in the beta frequency, there was no significant interaction (training × group) and no significant main effect of group across all parameters. However, significant main effects of training were found in all examined regions (F, C, T-L, T-R, and P/O). Particularly, significantly higher values during the post- compared to the pre-training bouts were found in CIb in all regions with p < 0.001, in CIs in the F (p=0.01), C, and P/O (p=0.02) regions, in DL in the C (p=0.01), T-R (p=0.002), and P/O (p=0 < 0.0001) regions, and in the RL group in the C (p=0.01), T-R and P/O (p < 0.001) regions (Table 1). Regarding the Δ pre-post training bouts, a significantly lower increase was observed in the CIs compared to CIb group in the T-R region (p=0.01). In the same way, CIb showed a trend toward nonsignificant higher increases compared to DL and RL in frontal (ES=0.7 and 0.6, respectively) and T-L (ES=0.8 and 0.5, respectively) regions and compared to CIs in T-R (p=0.8) and P/O (p=0.6) regions (Figure 4).

### Gamma

Equally, the analysis of gamma frequency yielded significant main effects of training in all examined regions (F, C, T-L, T-R, and P/O), with no significant interaction (training × group) and no significant main effect of group across all parameters. Particularly, significantly higher values during the post- compared to the pre-training bouts were found in CIb in all regions (p range between 0.0001 and 0.003), in CIs in the F (p=0.01), C (p=0.03) and P/O (p=0.04) regions, in DL in the C (p=0.01), T-R (p=0.01) and P/O (p=0.004) regions, and in the R group in the F (p=0.004), C (p=0.01), T-R (p=0.001) and P/O (p=0.002) regions (Table 1).

**FIG. 5:**
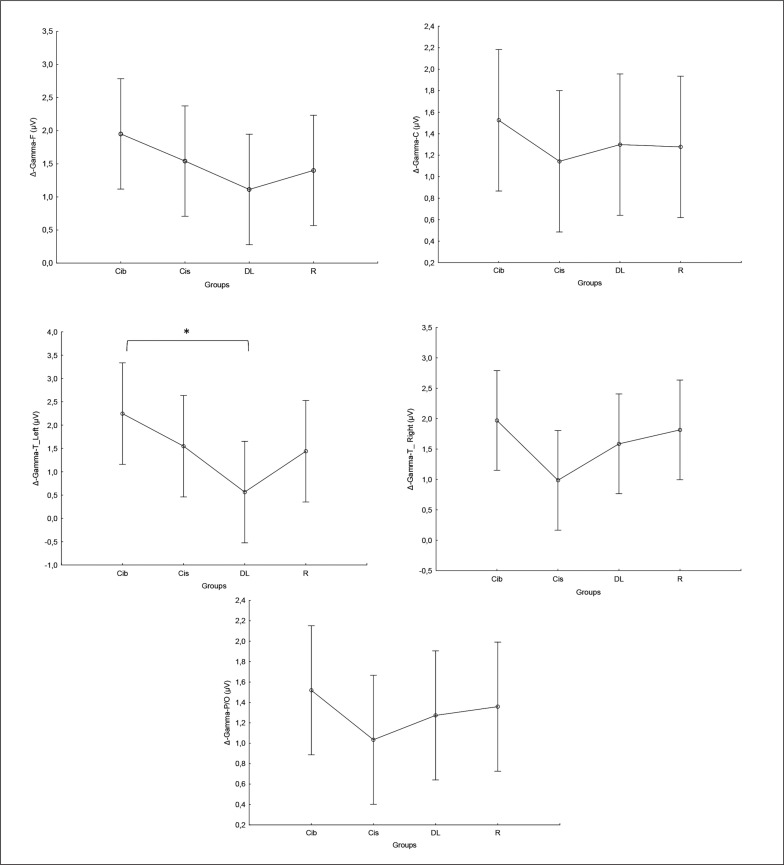
The acute effect of weightlifting training bouts on Δ (pre-post bouts) Gamma frequency recorded at the frontal, fronto-central, temporal (Left and right) and parieto-occipital regions. a: significant difference between groups at p < 0.05; Frontal (F), Central (C), temporal left (T_Left), temporal right (T_right) and parieto-occipital (P/O) regions; Contextual interference blocked (CIb), Contextual interference serial (CIs), Differential Learning (DL), Repetitive (R) motor learning models.

Regarding the Δ pre-post training bouts, significantly lower increases were observed in the DL compared to CIb group in the T-L region (p=0.03). In the same way, CIb showed a trend toward non-significant higher increases compared to DL in frontal T-L (ES=0.8) regions and compared to CIs in T-R (p=0.6) and P/O (p=0.5) regions (Figure 5).

### Resting HRV

The acute effect of the weightlifting training bouts on the HRV time and frequency domains parameters are presented in Table 2.

**TABLE 2:** The acute effect of the weightlifting training bouts on HRV’s time and frequency domains parameters

	Pre-training bout				Post-training bout				ANOVA 2 ways		
	CIb	CIs	DL	R	CIb	CIs	DL	R	Interaction Group × Training (F, p value, ηp^²^)	Group effect (F, p value, ηp^²^)	Training effect (F, p value, ηp^²^)
**HRV’s time domain (N ± SD)**											
HR-min	59.76 ± 9.09	62.05 ± 11.46	61.68 ± 8.78	63.05 ± 10.92	72.07 ± 11.83^a^	72.45 ± 12.90^a^	78.39 ± 11.17^a^	80.11 ± 13.42^a^	(F=2.61, p=0.06, ηp^²^= 0.12)	(F=0.93, p=0.43, ηp^²^= 0.05)	(F=190.48, p=0.0000, ηp^²^= 0.76)
HR-max	80.29 ± 15.13	80.94 ± 13.78	78.47 ± 14.06	80.19 ± 14.98	86.50 ± 12.84	86.48 ± 13.53	95.57 ± 11.66^a^	92.87 ± 15.68^a^	(F=3.27, p=0.03, ηp^²^= 0.14)	(F=0.35, p=0.79, ηp^²^= 0.02)	(F=46.17, p=0.0000, ηp^²^= 0.44)
HR-mean	66.74 ± 10.27	68.56 ± 11.89	67.68 ± 9.89	69.62 ± 12.76	77.89 ± 12.29^a^	77.85 ± 12.94^a^	84.47 ± 11.11^a^	85.23 ± 14.24^a^	(F=3.28, p=0.03, ηp^²^= 0.14)	(F=0.70, p=0.56, ηp^²^= 0.03)	(F=178.15, p=0.0000, ηp^²^= 0.75)
Mean RR	925.85 ± 144.78	906.10 ± 161.04	908.42 ± 131.80	893.93 ± 165.93	790.56 ± 122.90^a^	794.81 ± 140.70^a^	725.75 ± 107.10^a^	729.09 ± 156.41^a^	(F=2.14, p=0.11, ηp^2^ = 0.10)	(F=0.46, p=0.71, ηp^²^= 0.02)	(F=186.74, p=0.0000, ηp^²^= 0.76)
HRV-SDNN	59.66 ± 21.05	58.05 ± 25.48	53.08 ± 18.06	56.73 ± 18.32	37.30 ± 19.01^a^	38.26 ± 20.72^a^	30.78 ± 25.75^a^	24.70 ± 11.56^a^	(F=0.92, p=0.44, ηp^²^= 0.05)	(F=0.90, p=0.45, ηp^²^= 0.04)	(F=71.99, p=0.0000, ηp^²^= 0.55)
HR-RMSSD	57.41 ± 24.22	55.32 ± 22.99	52.86 ± 23.66	56.16 ± 23.32	38.15 ± 26.10^a^	39.68 ± 24.90^a^	27.30 ± 18.91^a^	25.21 ± 16.24^a^	(F=1.62, p=0.19, ηp^²^= 0.08)	(F=0.69, p=0.56, ηp^²^= 0.03)	(F=72.87, p=0.0000, ηp^²^= 0.55)
**Frequency domains and covariance (N ± SD)**											
HFn	0.41 ± 0.21	0.42 ± 0.18	0.49 ± 0.23	0.46 ± 0.19	0.40 ± 0.20	0.44 ± 0.22	0.33 ± 0.14^a^	0.41 ± 0.16	(F=2.53, p=0.07, ηp^²^= 0.11)	(F=0.12, p=0.95, ηp^²^= 0.01)	(F=4.05, p=0.04, ηp^²^= 0.06)
LFn	0.41 ± 0.22	0.40 ± 0.19	0.36 ± 0.18	0.39 ± 0.19	0.40 ± 0.20	0.41 ± 0.19	0.49 ± 0.12^a^	0.41 ± 0.14	(F=2.02, p=0.12, ηp^²^= 0.09)	(F=0.07, p=0.97, ηp^²^= 0.003)	(F=3.22, p=0.08, ηp^²^= 0.05)
LFHF	2.01 ± 2.90	1.37 ± 1.25	1.30 ± 1.39	1.27 ± 1.30	1.44 ± 1.18	1.39 ± 1.12	1.98 ± 1.46	1.33 ± 1.04	(F=1.22, p=0.31, ηp^²^= 0.06)	(F=0.42, p=0.74, ηp^²^= 0.02)	(F=0.05, p=0.83, ηp^²^= 0.001)
CVNN	6.40 ± 1.89	6.37 ± 2.51	5.82 ± 1.71	6.34 ± 1.63	4.58 ± 1.99^a^	4.67 ± 2.19^a^	3.97 ± 2.49^a^	3.29 ± 0.93^a^	(F=1.01, p=0.39, ηp^²^= 0.05)	(F=0.97, p=0.42, ηp^²^= 0.05)	(F=44.26, p=0.0000, ηp^²^= 0.43)

a : significant difference from pre to post; Frontal (F), Central (C), temporal left (T_Left), temporal right (T_right) and parieto-occipital (P/O) regions; Contextual interference blocked CIb, Contextual interference serial (CIs), differential learning (DL), Repetitive (R) motor learning models.

### Time domain parameters

There was no significant main effect of the group for any of the time domain parameters, while significant main effects of training were registered for all parameters, and a significant interaction (Time × group) was registered in HR-mean and HR-max. Particularly, significantly higher values of HR-min (p < 0.0001) and HRmean (p < 0.001) and lower values of mean RR (p < 0.0001), SDNN and RMSSD (p values range between < 0.0001 and 0.02) were found during the post- compared to the pre-training bouts in all groups. Higher values of HR-max were registered at post- compared to pre-training bouts only for the DL and R groups (p < 0.0001 and p=0.003, respectively) (Table 2).

**FIG. 6:**
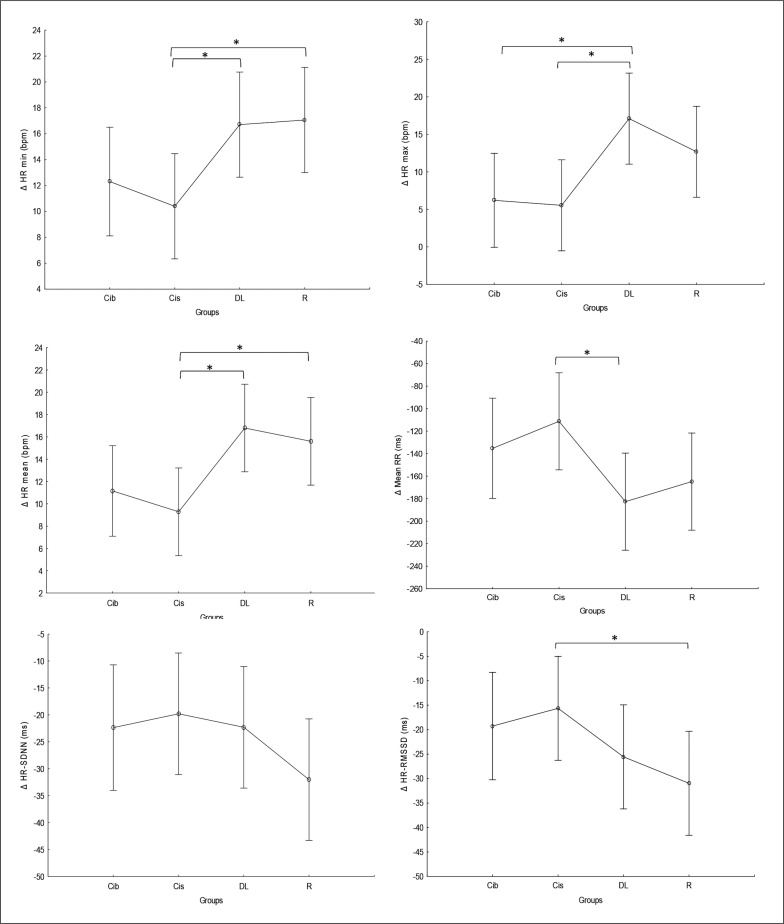
The acute effect of weightlifting training bouts on Δ (pre-post bouts) HRV´s time domain parameters. a: significant difference between groups at p < 0.05; Minimum heart rate (HR min); Maximum heart rate (HR max); Mean Heart Rate (HR mean); mean RR interval (Mean RR); Standard deviation of NN intervals (SDNN); Root mean square of successive differences (RMSSD); Contextual interference blocked (CIb), Contextual interference serial (CIs), Differential Learning (DL), Repetitive (R) motor learning models.

Regarding the difference between groups in terms of Δ pre-post training bouts (Figure 6), the DL group showed significantly more prominent increases in HR-min (p=0.03), HR-max (p=0.01) and HR-mean (p=0.01) and a decrease in mean RR (p=0.02) compared to the CIs. Additionally, more prominent increases in HR-mean (p=0.02) and HR-min (p=0.02) and a decrease in RMSSD (p= 0.04) were recorded in the RL group compared to CIs.

### Frequency domain parameters

There was no significant main effect of group or significant interaction (Training × group) in any of the tested parameters. Significant main effects of training were found for HFn, LFn and CVNN (Table 2), with lower CVNN values at post- compared to pre-training bouts in all tested groups (p values range between < 0.0001 and 0.02) and higher LFn values (p=0.03) and lower HFn values (p=0.001) at post-compared to pre-training bouts only in the DL group.

**FIG. 7:**
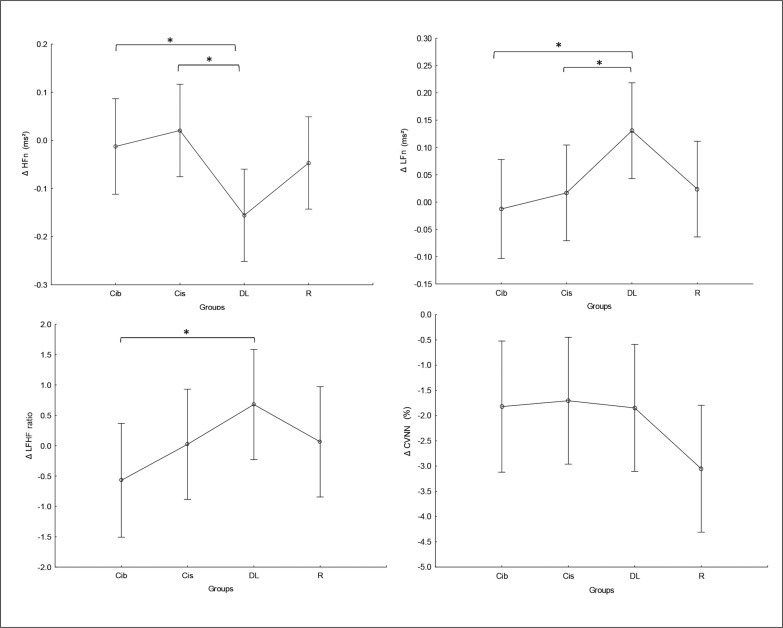
The acute effect of weightlifting training bouts on Δ (pre-post bouts) frequency domains and covariance. a: significant difference between groups at p < 0.05; HF, high-frequency power; Low-Frequency power (LF); Ratio of Low Frequency to High Frequency (LF/HF ratio); coefficient of variation (CVNN); Contextual interference blocked (CIb), Contextual interference serial (CIs), Differential Learning (DL), Repetitive (R) motor learning models.

Regarding the difference between groups in terms of Δ pre-post training bouts (Figure 7), the DL group showed significantly more prominent increases in LFn and decreases in HFn compared to the CIb (p=0.03 and 0.04, respectively) and CI groups (p=0.05 and 0.01, respectively). Likewise, a more prominent increase in LFHF was revealed in the DL group compared to CIb (p=0.05).

### HR zones of the weightlifting training bouts

**FIG. 8:**
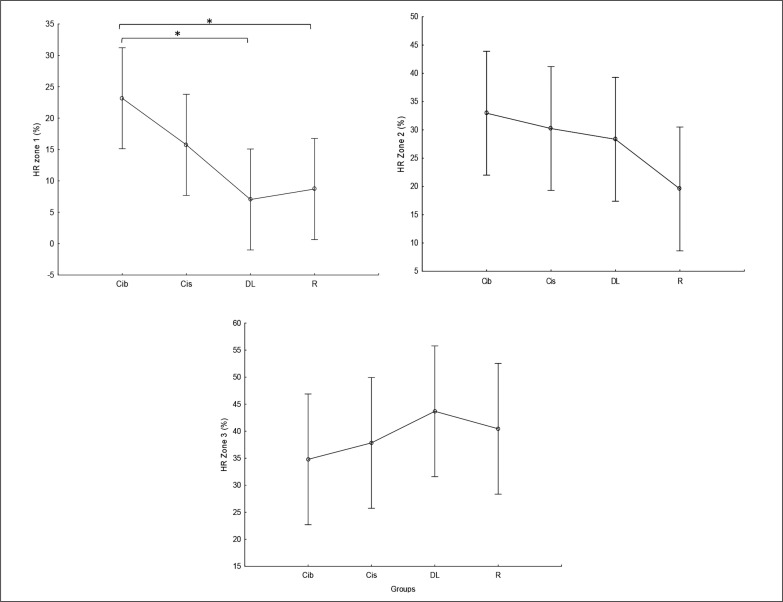
Proportion of the time spent in HR zones 1 to 3 during the weightlifting training bouts. a: significant difference between groups at p < 0.05; Contextual interference blocked (CIb), Contextual interference serial (CIs), Differential Learning (DL), Repetitive (R) motor learning models.

Times (in %) spent in HR zones 1, 2, and 3 during the weightlifting training bouts are presented in Figure 8. As this figure shows, less time was spent in HR zone 1 (< 60% of HR max) following DL and RL practices compared to CIb (p=0.01). In contrast, although not significant, during the DL practice, participants tended to spend more time in HR zone 3 (70–80% of HR max) compared to CIb (ES=0.5) and CIs (ES=0.4).

### Correlation between HRV and EEG-related parameters

The correlation coefficients between selected HRV and EEG responses to the weightlifting training bouts are presented in Table 3. As this table indicates, pooled HFn was significantly correlated with (i) beta and gamma frequencies in all tested brain regions except the frontal lobe (r=0.3 with p range between 0.01 and 0.03) and (ii) alpha frequencies only in the frontal lobe (r=0.3, p=0.03). Additionally, the LFHF ratio was shown to significantly correlate with alpha and beta frequencies in the frontal and P/O lobes, respectively (r=-0.3 and p=0.03). Looking at the correlation in response to each motor learning model, significant negative correlations were found between SDNN and alpha P/O and beta F with r=-0.5 and p=0.04.

**TABLE 3:** The correlation coefficients between selected HRV and EEG responses to the weightlifting training bouts.

		HRV’s time domain			Frequency domains and covariance			
		Mean R-R	SDNN	RMSSD	HFn	LFn	LFHF	CVNN
Alpha frequency	Fp/F	r=-0.17 p=0.19	r=-0.06 p=0.65	r=0.002 p=0.99	**r=0.27** **p=0.03**	r=-0.17 p=0.18	**r=-0.27** **p=0.03**	r=0.02 p=0.87
	C	r=-0.15 p=0.24	r=-0.11 p=0.40	r=-0.05 p=0.71	r=0.22 p=0.08	r=-0.12 p=0.35	r=-0.18 p=0.17	r=-0.06 p=0.66
	T_links	r=-0.16 p=0.20	r=-0.15 p=0.23	r=-0.08 p=0.53	r=0.21 p=0.10	r=-0.15 p=0.25	r=-0.18 p=0.15	r=-0.10 p=0.42
	T_recht	r=-0.17 p=0.17	r=-0.13 p=0.29	r=-0.10 p=0.43	r=0.19 p=0.13	r=-0.11 p=0.39	r=-0.20 p=0.12	r=-0.09 p=0.50
	P/O	r=-0.19 p=0.14	r=-0.13 p=0.31	r=-0.07 p=0.57	r=0.23 p=0.07	r=-0.17 p=0.19	r=-0.22 p=0.08	r=-0.08 p=0.56
Beta frequency	Fp/F	r=-0.16 p=0.20	r=-0.06 p=0.62	r=-0.01 p=0.91	r=0.22 p=0.09	r=-0.11 p=0.39	r=-0.13 p=0.30	r=-0.004 p=0.97
	C	r=-0.05 p=0.69	r=-0.04 p=0.78	r=0.03 p=0.82	**r=0.31** **p=0.01**	r=-0.18 p=0.16	r=-0.22 p=0.08	r=-0.01 p=0.92
	T_links	r=-0.12 p=0.33	r=-0.15 p=0.25	r=-0.06 p=0.65	**r=0.28** **p=0.03**	r=-0.18 p=0.16	r=-0.21 p=0.10	r=-0.12 p=0.36
	T_recht	r=-0.10 p=0.45	r=-0.01 p=0.93	r=0.01 p=0.97	**r=0.29** **p=0.02**	r=-0.16 p=0.19	r=-0.24 p=0.06	r=0.03 p=0.84
	P/O	r=-0.07 p=0.60	r=-0.05 p=0.73	r=0.02 p=0.91	**r=0.32** **p=0.01**	r=-0.20 p=0.11	**r=-0.27** **p=0.03**	r=-0.01 p=0.91
Gamma frequency	Fp/F	r=-0.13 p=0.32	r=-0.02 p=0.89	r=0.01 p=0.94	r=0.18 p=0.16	r=-0.08 p=0.52	r=-0.03 p=0.79	r=0.03 p=0.79
	C	r=-0.01 p=0.92	r=0.07 p=0.56	r=0.10 p=0.43	**r=0.27** **p=0.03**	r=-0.15 p=0.24	r=-0.12 p=0.33	r=0.10 p=0.43
	T_links	r=-0.04 p=0.76	r=-0.01 p=0.93	r=0.05 p=0.71	**r=0.27** **p=0.03**	r=-0.17 p=0.19	r=-0.14 p=0.26	r=0.02 p=0.89
	T_recht	r=-0.08 p=0.54	r=0.11 p=0.39	r=0.09 p=0.47	**r=0.27** **p=0.03**	r=-0.17 p=0.19	r=-0.16 p=0.22	r=0.16 p=0.21
	P/O	r=0.03 p=0.81	r=0.14 p=0.27	r=0.16 p=0.22	**r=0.30** **p=0.02**	r=-0.18 p=0.15	r=-0.15 p=0.22	r=0.18 p=0.16

### RPE and mental demand

**FIG. 9:**
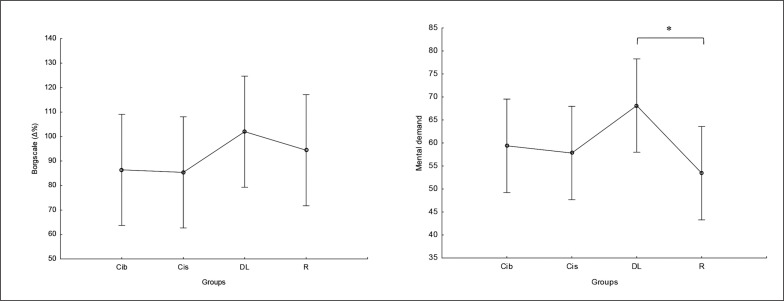
The acute effect of weightlifting training bouts on perceived exertion and mental demand. a: significant difference between groups at p < 0.05; Contextual interference blocked (CIb), Contextual interference serial (CIs), Differential Learning (DL), Repetitive (R) motor learning models.

There was no significant main effect of group on perceived exertion and mental demand. Post hoc comparison showed no significant differences between groups in RPE values, with only a trend for higher values in DL compared to CIb and CIs (ES= 0.5) (Figure 9). Regarding mental demand, post hoc comparison showed significantly higher values following DL compared to RL practice (p=0.04) and trends toward higher values compared to both CI practices ( ES ranging between 0.5 and 0.6) (Figure 9).

### Kinematic variables

**FIG. 10:**
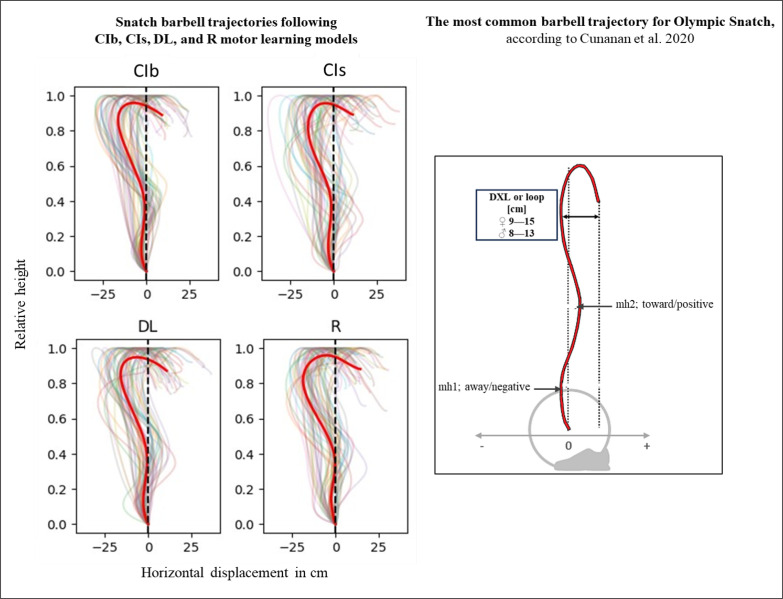
The acute effects of various weightlifting training bouts on technical efficiency based on barbel paths.

The acute effects of the weightlifting training bouts on technical efficiency based on barbel paths and selected barbel displacements are presented in Figure 10. The one-way ANOVA showed no significant main effect of the group in any of the tested parameters (peak vertical displacement; mh1, mh2 and DXL). In the same way, pairwise comparisons showed no significant differences between groups.

## DISCUSSION

The purpose of the present study was to assess the acute systemic effects of weightlifting training bouts following different motor learning models on neurophysiological responses and technical efficiency in novices. The main findings revealed increases in alpha, beta, and gamma frequencies following weightlifting training bouts, while theta frequencies remained unaffected. Notably, CIb practice resulted in increased frequencies across the alpha, beta, and gamma bands in 12 out of 15 brain regions (5 regions per band). Subsequently, RL practice showed increases in 10 out of 15 regions, and DL and CIs practice exhibited changes in 8 out of 15 regions. Regardless of the learning models, these changes in brain activity were accompanied by an alteration in HRV’s time domain parameters, characterized by increased HR- and decreased RR-related parameters, with more pronounced changes in DL compared to CI. In terms of HRV frequency domain parameters, only DL exhibited increased LFn and decreased HFn. HFn demonstrated positive correlations with beta and gamma frequencies across all tested brain regions except the frontal lobe. In the frontal lobe, this correlation was only significant with the alpha frequency. During the weightlifting training bouts, elevated HR zones (≥ HR zone 3) predominated with DL showing a tendency toward more extended periods in HR zone 3 compared to the other models. Following the weightlifting training bouts, the DL model tended to exhibit higher perceived physical and mental exertions. Concerning the technical efficiency, assessed through selected time discrete and averaged parameters from the barbell trajectory, the current results indicate no significant advantage of one learning model over the other in novices.

Resting EEG brain activity can be measured shortly before and after an exercise intervention, which offers a noninvasive, reliable method to study temporary changes in electrocortical activity [78, 79]. Although strength exercise is assumed to induce task-related brain activity [80] and is recognized for its demand on focus and concentration [81, 82], increasing arousal and motivation [83], neurotransmitter release, and blood flow [84], potentially altering associated brain wave patterns, most studies examining the acute effect of exercise on electric brain activity most often focus on endurance-based exercise [42]. A recent systematic review by Hosang et al. [42] reported that out of the 47 included studies, only two [85, 86] evaluated brain activity responses (pre- vs. post-exercise) to strengthbased single joint exercises, while the remaining studies focused on cycling and running. Gwin and Ferrsis [85] compared the coherence between contralateral motor cortex EEG signals and lower-limb EMG in the beta and gamma bands during isometric vs. isotonic knee or ankle joint movements. However, the change from pre- to post-resting EEG activity was not controlled. In contrast, Brümmer et al. [86] investigated the change from pre- to post-exercise bouts. However, the explored exercise bouts focused on a strength-based exercise involving only a single joint, controlled by a small muscle group in a seated position (i.e., dynamometer wrist flexion) to minimize coordinative aspects. In our exploration of various databases and sources, we also identified two EEG-based studies on strength training [80, 83] that specifically investigated the electrical activity of the brain during the bench press. Maszczyk et al. [83] identified motivational patterns through frontal alpha asymmetry at an intensity of 60% 1RM and above, while Engchuan et al. [80] observed increases in beta and gamma frequency bands during the bench press. However, careful interpretation is warranted due to certain methodological considerations. In the study by Engchuan et al. [80], EEG activity was recorded from only one location (Fp1), sensitive to muscular and ocular artifacts. Additionally, EEG activity was solely recorded during exercise, marked by a broader range of electrical muscle activity (10–200 Hz), potentially influencing gamma bands (30.5–60 Hz). Conversely, Maszczyk et al. [83] employed automatic headcaps with 19 electrodes for EEG recording, but analysed EEG activity exclusively from two locations (F3 and F4). Additionally, it’s worth noting that resting EEG activity was recorded for only 15 seconds before each intensity [83], whereas the recommended recording duration for establishing a baseline resting state [87] and achieving reliable results, especially in lower frequency bands, is typically 5–10 minutes. While these studies offered preliminary insights into the acute effects of strength exercise on neurophysiological EEG-based responses, none conducted a comprehensive evaluation of the acute impact on resting brain activity within the context of a strength-dominated whole-body task. Furthermore, none explored the effects of combined coordination-strength-based whole-body tasks. The present study on the latter type of movement represents, therefore, a pioneering effort to comprehensively explore this research field.

The present EEG findings indicate that regardless of the adopted motor learning approach, significant increases in beta and gamma frequencies were found in both the central and P/O regions following the weightlifting training bouts. These findings align with those of Engchuan et al. [80] during bench press movements. Similarly, regardless of the adopted motor learning approach, the present findings report an increase in alpha frequency in the P/O region. An increase in alpha frequency is typically associated with memory consolidation [88], integration of different brain areas [89], and introspection [90], which appears to be particularly pronounced in the studied whole-body movement. According to the dual-mode theory of exercise-related affect [91] and considering limited cortical resources, it is suggested that acute whole-body exercise leads to a redistribution of brain activity, particularly in areas typically associated with preparing and performing motor commands, such as the motor and somatosensory cortex. The present findings only partially support this theoretical framework, specifically in relation to increased motor and somatosensory cortex activities. Indeed, alpha, beta, and gamma frequencies increased after most of the tested weightlifting bouts in the (i) parietal lobe, known to contain the primary somatosensory cortex crucial for spatial awareness and processing sensory information related to body position and movement; (ii) occipital lobe, which encompasses the primary visual cortex responsible for processing visual information from the eyes; and (iii) central lobe, incorporating the specific C3 and C4 electrodes capturing activity in the primary motor cortex (M1). However, the present findings did not fully support the redistribution of brain activity in response to strength-based exercise, as such frameworks could indicate decreases in other brain regions, such as the frontal lobe, previously suggested by the transient hypo-frontality hypothesis [92]. Indeed, none of the different frequency bands showed a significant decrease in the frontal lobe or in the temporal lobe in response to the present weightlifting exercise.

Particularly, a deeper look at the frontal alpha activities following each of the motor learning models indicates either a significant increase after the CIs model or a stagnation using the CIb, DL, and RL models. Thus, the hypo-frontality hypothesis, previously mentioned by some studies to explain the decrease in frontal alpha activity in response to some endurance-based exercises [86, 93], cannot be supported in response to the conditions presented with combined coordination-strength training. The notable increase in frontal alpha activity following the CI models might be reflective of the execution of cognitive functions such as attentional focus and recovery strategies, known to be a germane process to exercise [42, 94, 95]. Significant increases in central alpha activity have been previously suggested to reflect a decrease in neuro-connectivity from somatosensory afferents [96], as the central sites of the brain have been shown to overlay regions of the sensorimotor cortex that receive afferent feedback [97]. The present findings on alpha frequency did not align with this assumption and showed that the increases in the central region following CIb and RL practices were accompanied by elevated alpha P/O following both models, along with increased alpha T-R following RL. In addition to these increases following the CIb and RL models, alpha frequencies also exhibited increases in P/O following CIs and DL, along with an increase in frontal and T-R regions, respectively, following these two models. These findings are in line with previous studies in endurance-based training reporting enhanced alpha activity both during and post-exercise across the limbic lobe/system (deep in the temporal lobe) [98, 99]. It might be argued that such an increase in gamma activity during exercise results from the cortical inhibition caused by the brainstem and subcortical activation [42, 100, 101]. Taken together, the present study provided evidence that in the P/O region, alpha frequency can increase regardless of the motor learning models applied to whole body movements, while in the other brain regions, changes (increases or stabilizes) depend on the learning model used. These coordination-strength-related findings seem to align with findings from the majority of previous endurance-based studies reporting either an overall increase in alpha activity mainly across the frontal areas [102, 103], with left lateralization [104], central [105, 106] and parieto-occipital [86, 107, 108] regions of the brain, or an absence of significant change in specific regions such as the left prefrontal cortex [109] and the bilateral frontal and temporal or parietal cortex [110, 111].

Regarding the beta frequency band, previous endurance-based exercise revealed primarily increased values, followed by decreased values and no significant changes from pre- to post-exercise [42], with increased activity during and after cyclic endurance activities mainly identified across the frontal [109, 112], central [112], and parietal [113] cortex regions [86] and limbic area [98]. The present coordination-strength-related findings align more with approaches suggesting enhanced beta activity in response to physical exercise by showing that in addition to the abovementioned central and parieto/occipital beta increases registered regardless of the motor learning model, there was also an increase in the remaining tested region for CIb, in the frontal region for CIs, and in the T-R region for DL and RL. Enhanced beta waves from pre- to post-exercise across the sensorimotor areas, reflected in the present study by the increased central and P/O beta activities following all tested strength bouts, can be associated with movement planning and production [114], while the enhanced beta activity across the temporal lobe using CIb (both left and right regions), DL, and R (only the right region), might reflect emotional arousal [115] in response to coordination-strength bouts. However, high-resolution EEG-based studies focusing on the limbic area, known to reflect affection [116], and measuring affective responses to exercise performed using different motor learning approaches are needed to test this hypothesis.

Concerning gamma activity, only a limited knowledge base is available regarding its response to physical exercise. According to the recent systematic review by Hosang et al. [42], out of the 47 included studies, only five explored this frequency band, with two of them testing pre- and post-exercise change and none of them focusing on strength or coordinative-strength exercises. Again, contradictory results between an increase [112] and a decrease [117] in response to endurance exercises in different contexts were reported. Similar to the beta frequency, the present coordination-strength-based findings align more with approaches suggesting enhanced gamma activity in response to physical exercise and showed that in addition to the abovementioned central and parieto/occipital beta increases registered regardless of the motor learning model, there was also an increase in the remaining tested region for CIb, in the frontal region for CIs, and in the T-R region for DL and RL. Such increases in gamma activity in response to strength and/or endurance dominant exercise can be linked to the increased arousal that is germane to exercise performance [118, 119]. However, due to the limited knowledge in this field, further studies measuring gamma activity in response to strength and coordination strength-based exercises are warranted to arrive at firm conclusions.

Lastly, it should be highlighted that the present coordinationstrength-based findings showed no change between pre- and post-exercise in terms of theta frequency in any of the tested regions. In response to endurance-based exercise, previous studies showed mainly a significant change from pre- to post-exercise, with either a decrease across the left prefrontal cortex [109] and the central and parietal cortices [106] or an increase in the bilateral prefrontal cortex [112]. Only one study showed no significant changes in this frequency band. Decreases in theta activity have been suggested to be indicative of impaired cognitive performance immediately after exercise, as well as the absence of relaxation [109, 119]. It can therefore be argued that the absence of a significant decrease in theta activity from pre- to post-weightlifting bouts in the present study can be reflective of reduced cognitive impairment and enhanced relaxation in response to this specific type of exercise. Such an assumption provides an advantage of strength over some endurance-based training (i.e., previously shown to decrease theta activity in different regions of the brain) in terms of exercise-induced low-frequency bands. However, this assumption needs to be corroborated by a range of coordination-strength-based exercises.

In summary, the present study provides a more comprehensive understanding of the profound effect of coordination-strength exercise, specifically weightlifting exercise, on EEG-based brain activity. In addition to the specific mechanisms associated with the different EEG frequency bands mentioned earlier, the relationship between exercise and brain activity in general has been previously associated with alterations in synaptic plasticity neurogenesis in the hippocampus in response to exercise [120]. The actions of signaling molecules such as brain-derived neurotrophic factor and insulin-like growth factor-1 may play pivotal roles in these processes [121–123]. Increased circulating cortisol immediately following exercise has also been shown to affect resting brain oscillatory activity post-exercise [124]. Beyond potential biochemical and hormonal influences, future research should explore mid and long-term effects, as well as individual and situational specificities, to comprehensively understand the complexity of these interactions [1].

To obtain a more comprehensive understanding of the neurophysiological responses to strength and coordination-strength exercise, the present study incorporated resting HRV measurements, known to mainly reflect the function of the autonomic nervous system (ANS) [125], in parallel with EEG recordings. A detailed analysis of the acute effect of coordination-based strength exercise on HRV-related parameters revealed an increase in HR-related parameters (e.g., min, max, and average) and a decrease in RR-related parameters (i.e., mean RR, HRVSDNN, and RMSSD), with more pronounced changes in DL. In terms of HRV frequency domain parameters, only DL exhibited increased LFn and decreased HFn. These changes suggest the existence of strong physiological strain and a connected regulation of the ANS throughout cardiac vagal control in response to the weightlifting training bouts. In general, HF-HRV is considered to be more prominently dominated by parasympathetic tone [126], while LF-HRV reflects brain activity involved in executive functioning, including working memory utilization [127, 128]. It seems therefore reasonable to assume that strength exercise combined with DL practice seems to reduce parasympathetic resting dominance through lowered vagal modulation and increased sympathovagal balance [129], as well as engage individuals in higher working memory utilization and executive function involvement. These suggestions align with the present results of the perceived mental demand, indicating a trend toward higher mental demand following DL compared to the other learning model (ES ranging between 0.5 and 0.6), which reached significance when compared to RL practice. The extent to which this is related to the increased interaction between the heart and brain, previously reported in DL in combination with rope skipping [47], requires further research. Additionally, more coordination-strength-based studies in different motor learning models are needed to corroborate these suggestions. The present effects of weightlifting-bouts exercise on HRV align with previous findings by de Paula et al. [129], who demonstrated that both aerobic- and strength-based exercise elicited an acute change in HRV characterized by increased HR, LF, and LFHF ratio, along with decreased RR interval and HF. However, this study focused only on individuals with autonomic dysfunction. In healthy adults, previous HRV studies in the field of physical exercise have predominantly focused on the chronic effects of various exercise training regimens on HRV parameters. Notably, strength training has been comparatively less explored in this context and has yielded controversial results [130]. Isometric handgrip training (IHG) and high-intensity strength training fail to induce HRV modifications [131, 132]. Whole-body resistance training in young women has been shown to significantly improve geometric indices [133]. Similarly, 8 weeks of two different resistance training methods (clustering vs. multisets) were found to increase RMSSD and decrease resting heart rate [134]. An increase in autonomically mediated heart rate complexity has also been registered after resistance training, reflecting increased parasympathetic and/or reduced sympathetic cardiac autonomic control [135]. Comparing the chronic effects of high vs. low resistance training intensity, Lin et al. [136] reported improvements in resting heart rate and specific HRV measures in high-intensity training compared to low-intensity training. Again, more strengthbased studies in different motor learning models are needed to corroborate these suggestions. Regarding the acute effect, a previous study showed a decrease in RR interval and SDNN and an increase in LF following rope skipping bouts, with a more pronounced effect in the DL group [46]. Lower HRV has been previously correlated with higher cognitive demand, defined by greater executive task strain and particularly sustained attention [137]. Accordingly, the more pronounced decrease in HRV parameters accompanied by the higher mental demand perception and the increase in LF power in response to DL practice registered in the present as well as in the rope skipping study support once more the hypothesis of higher cognitive demands after DL accompanied by specific parasympathetic involvement.

Concerning the correlation between EEG and HRV parameters, previous studies have mainly focused on simultaneously recording EEG and HRV in response to cognitive tasks [138–140]. These studies indicated that executive function is closely related to vagally mediated HRV parameters as a result of the involvement of inhibitory inputs from the prefrontal-subcortical inhibitory circuits to the heart via the vagus nerve [45]. Additionally, in several electrodes, beta relative power during the working memory task was found to be negatively correlated with resting HF-HRV [139]. These findings suggested HRV as a possible biosignal indicator reflecting brain activity related to working memory [139], executive control functions such as the inhibition of unwanted responses [141], and mental health status in general [44].

Despite these promising findings, limited number of studies that concurrently explore both EEG and HRV responses to physical exercise. For instance, John et al. [142] analyzed the correlation between both measurements during and following (i.e., short-term recovery) incremental exercise testing [142]. The findings showed moderate negative correlations between LF power (ECG) and beta and gamma frequencies (EEG) in total power and the frontopolar, temporal, and occipital lobes with LF power during exercise, which were reduced to only cover the temporal lobe of beta and gamma total spectra. These results are partially in line with the present one, showing a significant positive correlation between HFs and beta and gamma frequencies in 4 out of 5 brain regions (C, T-L, T-R, and P/O) and alpha frequency only in the frontal lobe. These results suggest links between cardiovascular strain induced by strength exercise and motor and sensory demands, followed by an executive function control requirement. A closer examination of the EEG-ECG correlation in each motor learning model revealed a negative correlation between SDNN and alpha frequency in the P/O region and beta frequency in the frontal lobe following DL practice. These findings indicate that the greater decrease in HRV-SDNN in response to coordination-strength exercise under DL practice was associated with greater brain activation in these specific regions.

It’s noteworthy that during DL practice, individuals were more engaged in the elevated HR zone (i.e., HR zone 3) and less engaged in the lower HR zone (i.e., HR zone 1) compared to other motor learning models. This heightened HR during DL practice may contribute the trend toward higher perceived exertion following the same training bouts compared to the other practice models (ES = 0.5). These findings suggest an advantage of DL over other models in terms of improved cardiac autonomic modulation and cardiovascular benefits. This aligns with a previous study that reported greater cardiac benefits of high-intensity exercise compared to low-intensity exercise [136]. Notwithstanding this, it is crucial to direct future attention to the observed phenomenon wherein the learning approach leading to the highest increase in physiological and cognitive load—namely DL, together with the CIs presenting the second largest exercise variations—demonstrated the lowest number of significant EEG-related increases. This is noteworthy as such neuro-physiological responses are typically positively correlated [142]. These findings further underscore the interplay between highly coordinated, variable training, whether in combination with endurance [47] or strength training, and its influence on the complex relationship between the heart and brain in unprecedented ways. The potential implications of these findings may extend to the field of therapies, offering novel avenues for exploration in the future.

In terms of technical efficiency, a recent study by Cunanan et al. [143] examining the barbell kinematics of top athletes participating in the 2015 world and 2017 Pan-American weightlifting championships, reported that the most common barbell trajectory for snatch follows the “‘away-toward-away-toward’ pattern with a loop (the horizontal displacement from the most forward position to the catch position) ranging between 9 and 15 cm for male athletes. This pattern describes that during the lift, the bar first moves away from the body, then toward the body, then away and toward the body again, resulting in negative-positive-negative-positive displacements [41, 52, 76]. The results from this weightlifting study, conducted with novices, indicate that regardless of the motor learning models employed, the averaged barbell paths do not align with the recommended trajectory. Indeed, positive horizontal displacement values are observed only during the “catch phase” at the end of the movement. Similarly, none of the loop values fell within the recommended range of 9–15 cm. Specifically, the absolute DXL values ranged between 23 and 27 cm following the four learning models, with no significant difference between them.

According to the overload of cognitive working memory [26], a worst technical performance was expected following the DL practice due to the higher cognitive demand, evidenced by the more pronounced decrease in HRV, increase in LF, and higher mental demand perception. The absence of significant differences between the different practice models in terms of technical outcomes calls this theory into question. Additionally, the present findings suggest that regardless of the motor learning model, one training bout of 36 total weightlifting trials was not enough to approach the high-performance model of complex Olympic snatch movements, involving the entire body musculature. Whether a certain weight threshold must be surpassed or more substantial increase in exercise noise is necessary for more effective changes requires further research. In the context of classical repetition-based motor learning, higher total trial numbers and/or longer practice durations have been previously recommended to elucidate differences in terms of technical efficiency between practice models [144, 145]. Whether individual characteristics dominate over motor learning models in relation to the acute effect of learning bouts on weightlifting technical efficiency, must be shown by future research. Nevertheless, future studies investigating individual responsiveness to such training bouts and employing artificial intelligence methods, such as machine learning and pattern recognition based on individual vs. motor learning models, are needed to corroborate this suggestion [146–148]. Similarly, to what extent the total trial numbers and/or longer intervention duration may affect motor learning advantages, requires further exploration in future studies.

### Strengths and limitations

This study represents a pioneering effort to assess the acute effects of weightlifting training bouts on neurophysiological response, perceived exertion, and mental demand while controlling the possible influence of adopting different motor learning models. Nevertheless, the study’s design precludes generalization, and it is crucial to acknowledge several limitations that should be considered when interpreting the present findings. Epistemologically, the scope of application is determined by the boundary conditions of the study. Within these constraints, the study offers recommendations for further research that could expand the findings in specific areas and overcome certain limitations.

Previous reports have suggested different functional significance of the two alpha ranges, namely, the lower component ranging between 7.5 and 9 Hz and the upper component ranging between 9.5 and 12.5 Hz [149], as well as the two beta ranges (beta 1 ranging between 13 and 20 and beta 2 ranging between 21 and 30 [150]). For instance, Abhang et al. [118] indicated that low beta waves (beta 1) are mainly associated with focused concentration, while beta 2 waves are more linked to increases in energy and performance. Hence, computing the average alpha or beta power across all electrodes may yield imprecise results. Furthermore, in the present study, the EEG system and the Polar H10 sensor were not digitally synchronized. This limitation restricted our analysis to calculating correlations between averaged values recorded simultaneously during the 5-minute resting states and precluded the possibility of conducting cross-correlation analyses. Another limiting factor in the present study pertains to the number of electrodes employed for brain activity localization. A recent review, commissioned by the International Federation of Clinical Neurophysiology (IFCN), has suggested that a minimum of 48–64 electrodes may be necessary to achieve a high level of confidence in this localization. Consequently, future studies in the field of applied neurophysiology may benefit from employing a more rigorous methodology to confirm the precise anatomical localization of exercise effects on the brain.

## CONCLUSIONS

Weightlifting training bouts show the potential to increase alpha, beta, and gamma frequencies in the majority of the tested brain regions, accompanied by an increase in HR and a reduction in RR-related parameters. While the impact of the adopted motor learning models on brain activity remains somewhat ambiguous, a more pronounced effect is observed in the DL model on both time and frequency domain HRV parameters. Especially when coupled with the lowest number of significant EEG changes, the DL approach once again confirms its extraordinary impact, not only on the brain but also on its interaction with the heart. These findings provide preliminary support for the acute neurophysiological benefits of strength-based exercise, especially when performed using a DL model. However, further studies encompassing a broader range of coordination-strength-based exercises are warranted to corroborate these suggestions. The use of stressrelated EEG features combined with correlated HRV features appears to offer a more comprehensive understanding of neurophysiological responses to human movement under stress, potentially enhancing the detection and monitoring of related adaptations following acute and/or chronic interventions with different motor learning approaches.
